# Do Not Get Your Uterus Twisted: A Case Report of a 180-Degree Torsion of Term Gravid Uterus and a Review of the Literature

**DOI:** 10.7759/cureus.62194

**Published:** 2024-06-11

**Authors:** Katie Boehm, Mariam Gheissari, David Crownover, Amanda Frugoli

**Affiliations:** 1 Graduate Medical Education, Family Medicine, Community Memorial Healthcare, Ventura, USA; 2 Graduate Medical Education, Obstetrics, Community Memorial Healthcare, Ventura, USA; 3 Pacific Inpatient Physicians, Community Memorial Hospital, Ventura, USA; 4 Graduate Medical Education, Community Memorial Hospital, Ventura, USA

**Keywords:** emergency obstetric care, external cephalic version, labor complication, fetal intolerance of labor, posterior hysterotomy, cesarean section (cs), gravid uterus, uterine torsion

## Abstract

Gravid uterine torsion less than 45 degrees is a common phenomenon of the third trimester. Torsion greater than 45 degrees represents a rare, pathologic, and obstetric emergency. The rotation of the uterus on a longitudinal plane can result in vascular compromise, and it has potential for catastrophic maternal-fetal complications. We report the case of a 22-year-old G3P1011, third pregnancy with history of one full-term live newborn, one spontaneous abortion, and presented at 38 weeks gestation with complaints of abdominal pressure and recurrent transverse fetal presentation. She underwent an external cephalic version (ECV), which resulted in fetal distress necessitating an emergency cesarean section. After successful delivery of the live newborn, an inspection of the uterus identified a uterine torsion of 180 degrees with delivery through a posterior hysterotomy incision. She had no postoperative complications and carried a subsequent pregnancy to term that was delivered via repeat cesarean section five years later. Gravid uterine torsion should be included in the differential diagnosis for patients presenting with abdominal pain and fetal intolerance to labor. A higher suspicion should be held for patients with a known history of uterine abnormalities or those having undergone an ECV. Our case also highlights a safe repeat cesarean section after this rare complication and brief narrative review of existing literature on this rare obstetrical emergency.

## Introduction

Uterine torsion is an uncommon occurrence. In the setting of gravid uterine torsion greater than 45 degrees, urgent to emergent operative intervention is usually required. The prevalence of this condition is unknown as much of the published data is limited to case reports. We conducted a literature review by searching PubMed for case reports and published material containing the terms “uterine/ uterus torsion” and “external cephalic version" and provided a narrated review.

Case reports commonly note pathological uterine torsion of at least 180 degrees (range from 45 to 720 degrees) about the uterocervical junction, and approximately two-thirds of cases occur in dextrorotation [1]. Pathological uterine torsion is typically diagnosed during the third trimester; however, it has been documented as early as the sixth gestational week [2]. The underlying risk factors of uterine malformations, leiomyomas, and abnormal fetal presentations have been cited most frequently. Other associated factors such as placental anomalies, pelvic tumors, congenital deformities, uterine adhesions, external cephalic version (ECV), and trauma have been described [2-4].

Patients typically present with abdominal pain, hemodynamic shock, and/or fetal distress. The differential diagnosis for this constellation of symptoms in the third trimester includes placental abruption, uterine rupture, peritonitis, and torsion of a pelvic tumor.

The pathophysiology of pathological gravid uterine torsion has not been well-elucidated. While it can occur in nongravid individuals, the majority of the cases described have been in gravid patients, suggesting that the component of pregnancy and associated uterine growth is a significant risk factor for uterine torsion. Another proposed contributing factor is the physiologic increase in the relaxin hormone during pregnancy that allows for the relaxation of the myometrium, separation of the symphysis pubis, and cervical softening. This tissue laxity can increase mobility around the uterocervical axis and contribute to gravid uterine torsion. However, this does not explain episodes of non-gravid uterine torsion. Moreover, it is possible that progesterone could be the culprit or contributor as recent studies have identified that progesterone can affect gene expression that results in uterine relaxation by repression of contraction-associated proteins [5]. It is unclear, and more research would be required to determine if the progesterone link would better explain uterine torsion in nonpregnant patients.

Gravid uterine torsion is more commonly observed in veterinary medicine among quadrupeds such as cattle, where the gravity-dependent uterus enlarges and stretches perpendicularly to the horizontal axis of the vagina and cervix [3,4]. Torsion is thus noted to occur at the intersection between the fixed vaginal segment and the mobile uterus. This proposes another mechanism for torsion, related to a prolonged all-fours posture.

The pathological sequelae of gravid uterine torsion include stenosis and compromise of the uterine vasculature with subsequent poor placental perfusion. This is noted to cause cyanotic changes and engorgement of the uterine vessels. Decreased placental perfusion can lead to abruption, fetal distress, and, ultimately, fetal demise without emergency delivery. Of the documented cases, a maternal mortality of 13%, and a fetal mortality of 18% was reported [6].

The onset of maternal hemodynamic shock or fetal intolerance of labor (fetal bradycardia or uterine tachysystole) often necessitates emergent cesarean section, whereby uterine torsion is discovered as an incidental finding intraoperatively. The diagnosis of uterine torsion is commonly made intraoperatively at the time of emergent cesarean section. There is some literature that supports the use of imaging such as ultrasound and MRI to make the diagnosis preoperatively. MRIs obtained in non-emergent settings note radiographic changes in the upper vaginal wall, from the standard “H” configuration to an “X” shape and ultrasound to confirm placental location and fetal lie [7]. These imaging modalities are not appropriate in emergent settings and likely account for the majority of cases being identified intraoperatively.

In the majority of cases, the fetal intolerance of labor or development of maternal hemodynamic shock results in emergent cesarean section. The discovery of a torsed uterus is usually identified after posterior hysterotomy for delivery [8]. Research is necessary to help determine an earlier detection strategy, ensure the safety of repeat pregnancies, and identify the best mode for delivery.

## Case presentation

We present a case of a 22-year-old G3P1011 with no significant past medical history who presented for her routine prenatal visit at 38 weeks with complaints of intermittent abdominal pressure. Her obstetrical history included a normal spontaneous vaginal delivery at 40 weeks gestation without complications, and a first trimester spontaneous abortion. She received routine prenatal care throughout her current pregnancy. She complained of episodes of dizziness during her pregnancy, which was attributed to her occupation as a fieldworker, involving daily bending at the waist to pick strawberries from the vine at ground level. The patient sustained a ground level fall onto her back and buttocks at 26 weeks gestation, resulting in low back and pelvic pain. At the time of her fall, she denied vaginal bleeding, abdominal pain, or contractions, and she received symptomatic pain management. At 31 weeks gestation, she was diagnosed with gestational diabetes mellitus, which was well-controlled with her diet for the remainder of her pregnancy.

When the patient presented at 38 weeks, she complained of intermittent “pressure” and lower pelvic pain, but she denied any associated vaginal bleeding, contractions, or loss of amniotic fluid. An ultrasound revealed an unstable fetal lie, alternating between transverse and breech positions. An ECV was performed and resulted in a cephalic position; however, the head was poorly engaged in the pelvis. The patient did not require neuraxial analgesia, and she tolerated the procedure well. A reactive fetal heart tracing was noted immediately after the procedure.

One week later, the patient presented for a follow-up appointment at 39 weeks gestation. On ultrasound, the fetus was again noted to be in a transverse position. The patient elected for a repeat ECV, and the fetus again converted to the cephalic position with poor head engagement. After the procedure, however, fetal bradycardia was documented at 50 bpm. Maternal vital signs were stable, and a cervical exam revealed a multiparous cervix with the external os dilated to 2 cm but a closed internal os.

An emergency cesarean section was performed because of the fetal bradycardia. A low-transverse incision was made, and the newborn was delivered from a breech position. The newborn was a vigorous and crying female, with appearance, pulse, grimace, activity, and respiration (APGAR) score of 8 and 9 at one and five minutes, respectively, and had a birth weight of 3,131g. The arterial cord gas pH was 7.1 and within normal limits. Only after delivery of the newborn was the uterus recognized to have undergone levorotation of approximately 180 degrees around the cervicouterine axis (Video 1). Once normal anatomy was restored, it was noted that the incision had been made on the posterior lower uterine segment.

**Video 1:** Torsion of Gravid Uterus with Posterior Hysterotomy

Anatomical inspection revealed a grossly normal-appearing uterus in both architecture and color. The fallopian tubes and ovaries appeared normal; however, the vasculature of the right adnexa was stretched across the posterior surface of the uterus (which was then most anterior because of the 180 degrees of torsion) and was engorged. The placenta appeared normal, was manually extracted, and the uterus was cleared of all clots and debris. The uterus was then exteriorized, and the torsion was reduced. The posterior hysterotomy was repaired with a 0-vicryl suture in a running-locked fashion. A second imbricating layer was used to obtain hemostasis. The uterus was returned to the abdomen, and the fascia, subcutaneous tissue, and skin were closed in the usual fashion. The patient was taken to the postanesthesia care unit in stable condition. The estimated blood loss was 700 mL. The patient had an uncomplicated postoperative hospital course and was discharged home with her newborn on postoperative day three.

She was able to have a second child with repeat cesarean section without complications five years later.

## Discussion

Gravid uterine torsion remains a rare phenomenon, with much of the medical literature existing in case reports. Case reports commonly note pathological uterine torsion of at least 180 degrees (ranging from 45 to 720 degrees) about the uterocervical junction, and approximately two-thirds of cases occur in dextrorotation [1]. We conducted a literature review of recent studies that included 27 case reports of gravid uterine. Unfortunately, fetal or neonatal demise was noted in three out of the 27 cases. This is a higher incidence than the USA-recorded rate of stillbirths of 5.89 [9].

After reviewing the current literature and existing case reports on uterine torsion, we have identified some proposed risk factors that appear more common [10-30]. Half of the case reports had some structural anomaly reported, such as leiomyomas, pelvic adhesions, or ovarian cysts, lending credence to the theory that anatomic abnormalities are a significant risk factor [9]. Our patient sustained a torsed term gravid uterus without any notable leiomyomas, placental anomalies, adhesions, or tumors, suggesting that her case was more unusual than those previously reported.

One risk factor identified in our patient was the malpresentation of the fetus in a transverse lie leading to an ECV. There were two other cases whereby uterine torsion was diagnosed following an ECV [2,4,10,11]. The ECV cannot be excluded as an underlying cause of uterine torsion; however, there is no direct testing to substantiate a direct cause-and-effect correlation. ECVs are routinely performed safely. A 2008 meta-analysis of ECV-related risks identified that of 84 studies (12,955 subjects) involving ECV performed after 36 weeks, the rate of serious adverse maternal and fetal outcomes was 6.1%. This included events such as transient fetal heart rate changes (4.7%) and emergency cesarean delivery (0.4%) [11]. Uterine torsion had not been established as an identified risk [4,10,11]. The American College of Obstetricians and Gynecologists (ACOG) proposes that the use of neuraxial anesthesia for ECV, used in combination with tocolytic therapy, can be a “reasonable intervention” to increase the success rate [31]. In our patient, the initial ECV was successful without neuraxial analgesia. Therefore, the repeat ECV was also performed without neuraxial anesthesia.

Another risk factor that may have predisposed our patient to a gravid uterine torsion was her occupation as an agricultural laborer. This is a job that requires a significant portion of time in a forward fold posture with a dependent uterus. Although the biomechanics for uterine torsion among quadrupeds has been described, similar occurrences outside of veterinary medicine do not exist in the literature [32]. 

The most common clinical presentation of uterine torsion cited in the literature is abdominal pain, followed by fetal heart rate-tracing abnormalities with the onset of normal labor. The majority of uterine torsion cases are diagnosed intraoperatively and often after a posterior hysterotomy, with only four cases recognized before incision. It is commonly the emergent nature of the cesarean that delays recognition of the posterior incision, as was the case with our patient.

Few case reports include data on subsequent successful pregnancies; however, it is unknown how many are lost to follow-up. Patients who have a history of uterine torsion are generally counseled to either avoid subsequent pregnancies or undergo a scheduled repeat cesarean section. Little research exists on the safety of vaginal births after cesarean sections following the term gravid uterine torsion. According to the ACOG practice bulletin entitled Vaginal Birth After Cesarean Delivery, “uterine rupture or dehiscence associated with a trial of labor after cesarean (TOLAC) results in the most significant increase in the likelihood of additional maternal and neonatal morbidity” [33]. Most prior cesareans involve anterior hysterectomies of the lower uterine segment. There are no large datasets regarding the risk of uterine rupture or dehiscence with posterior hysterotomy. Therefore, the decision was made to have a scheduled repeat cesarean at 39 weeks gestational age. The patient had an onset of labor before this and, therefore, underwent a repeat cesarean section before her scheduled date. In this regard, our patient is unique in that she was able to carry a future pregnancy to term and deliver without any adjunctive surgical interventions at the time of her primary cesarean section. One intervention that can be considered during the primary cesarean section is to attempt to stabilize the uterus prophylactically by plicating the round ligaments [30].

Finally, because of the posterior and anterior hysterotomies, there remains the concern for increased risk of placenta accreta and third-trimester uterine rupture before labor. In a systematic review, the rate of “placenta accreta spectrum” increased from 0.3% in women with one previous cesarean delivery to 6.7% for women with five or more cesarean deliveries [34]. Therefore, now that the patient has had two previous cesarean deliveries, her risk of placenta accreta spectrum has likely increased. However, it is unknown how having two uterine incisions in separate uterine locations alters this risk.

Further understanding of uterine torsion will require more research. It is unknown if the rarity of the condition is truly because of a low incidence or whether it is underreported. Future research can help in further understanding and identifying possible preventable risk factors. It is important to keep uterine torsion in the differential for a gravid patient presenting with abdominal pain [35]. Education surrounding intraoperative recognition and including observing the normal anatomy intraoperatively in patients undergoing cesarean section could decrease the incidence of posterior hysterotomy.

## Conclusions

Gravid uterine torsion should be included in the differential diagnosis for patients presenting with abdominal pain and fetal intolerance to labor. A higher suspicion should be held for patients with a known history of uterine abnormalities or those having undergone an ECV. An increase in physician awareness and education could improve intraoperative recognition and reduce the incidence of posterior hysterotomy. More reporting and research is required to extrapolate further regarding the maternal and fetal risks of adverse outcomes in current and future pregnancies. We present this case to add to the limited research on the rare obstetric complication.
